# 821. Changes in Cleaning Practices and Non-conventional Personal Protective Equipment Use due to SARS-CoV-2 and Association with Increases in Multi-drug Resistant Organism Cases

**DOI:** 10.1093/ofid/ofab466.1017

**Published:** 2021-12-04

**Authors:** Medora L Witwer, Susan E Kline, Patricia Ferrieri, Samantha Saunders, Ginette Dobbins, Kari Gand, Alison Galdys

**Affiliations:** 1 University of Minnesota Medical Center, St. Paul, Minnesota; 2 University of Minnesota, Minneapolis, MN; 3 University of Minnesota Medical School, Minneapolis, Minnesota; 4 M Health Fairview, Minneapolis, Minnesota; 5 University of Minnesota Health, Minneapolis, MN

## Abstract

**Background:**

During the pandemic of severe acute respiratory syndrome coronavirus 2 (SARS-CoV-2), policy at a Minnesota hospital changed to state that environmental services would not clean rooms of patients with confirmed or suspected SARS-CoV-2 infections, requiring nursing staff to perform these duties. Investigation of a cluster of carbapenem-resistant Enterobacterales (CRE) in patients hospitalized in the same or adjoining rooms on the medical intensive care unit (MICU) raised concern over whether SARS-CoV-2 cleaning practices and non-conventional personal protective equipment (PPE) use led to transmission of multi-drug resistant organisms (MDROs).

**Methods:**

Infection Prevention conducts passive surveillance for MDRO acquisition in inpatient units. Passive surveillance of SARS-CoV-2 was performed early in the pandemic. Active surveillance SARS-CoV-2 testing on admission was initiated in July 2020 and active surveillance testing for admitted patients every 7 days was initiated in December. Incident cases of vancomycin-resistant Enterococcus (VRE), extended-spectrum-β-lactamase-producing organisms (ESBL), methicillin-resistant *S. aureus* (MRSA), and CRE were determined for hospitalized patients between March 1, 2020 and February 28, 2021, excluding patients with infection on admission. Rates of hospitalized patients testing positive for SARS-CoV-2 per 100 patient days were compared to rates of patients testing positive for VRE, ESBL, MRSA, and CRE per 100 patient days respectively. The same rate comparisons were completed for the MICU. Using the F-Test Two-Sample to determine variance, the Two-Sample T-test assuming unequal variances was applied to each comparison.

**Results:**

Correlation was significant between rates of SARS-CoV-2 and VRE (*p< 0.005*), ESBL (*p< 0.005*), MRSA (*p< 0.005*), and CRE (*p< 0.005*) (Table 1). MICU correlation was significant between rates of SARS-CoV-2 and VRE (*p< 0.005*), ESBL (*p< 0.005*), MRSA (*p< 0.005*), and CRE (*p< 0.005*) (Table 2).

Table 1: Two-sample T-test results assuming unequal variances: Hospital COVID rates per 100 patient days vs. rates of incident positive tests for VRE, ESBL, MRSA, and CRE per 100 patient days

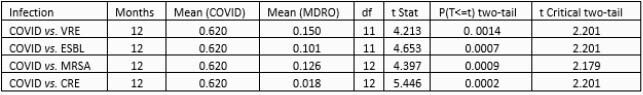

Table 2: Two-sample T-test results assuming unequal variances: MICU COVID rates per 100 patient days vs. rates of incident positive tests for VRE, ESBL, MRSA, and CRE per 100 patient days

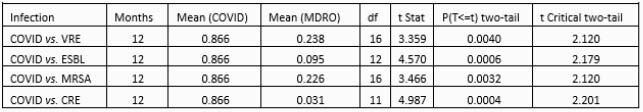

**Conclusion:**

The relationships between the rates of SARS-CoV-2 and four MDROs were statistically significant. It can be inferred from this data that changes in hospital cleaning and non-conventional PPE use may have led to an increase in transmission of MDROs in this facility.

**Disclosures:**

**All Authors**: No reported disclosures

